# Beneficial Effect of *Bidens pilosa* on Body Weight Gain, Food Conversion Ratio, Gut Bacteria and Coccidiosis in Chickens

**DOI:** 10.1371/journal.pone.0146141

**Published:** 2016-01-14

**Authors:** Cicero L. T. Chang, Chih-Yao Chung, Chih-Horng Kuo, Tien-Fen Kuo, Chu-Wen Yang, Wen-Chin Yang

**Affiliations:** 1 Department of Veterinary Medicine, College of Veterinary Medicine, National Chung-Hsing University, Taichung 402, Taiwan; 2 Institute of Plant and Microbial Biology, Academia Sinica, Taipei, Taiwan; 3 Agricultural Biotechnology Research Center, Academia Sinica, Taipei, Taiwan; 4 Department of Microbiology, Soochow University, Taipei, Taiwan; 5 Department of Aquaculture, National Ocean University, Keelung Chung, Taiwan; 6 Institute of Pharmacology, Yang-Ming University, Taipei, Taiwan; 7 Department of Life Sciences, National Chung-Hsing University, Taichung 402, Taiwan; University of New England, AUSTRALIA

## Abstract

In the interests of food safety and public health, plants and their compounds are now re-emerging as an alternative approach to treat gastrointestinal diseases in chickens. Here, we studied the impact of the edible medicinal plant, *B*. *pilosa*, on growth performance, gut bacteria and coccidiosis in chickens. First, we found that *B*. *pilosa* significantly elevated body weight gain and lowered feed conversion ratio in chickens. Next, we showed that *B*. *pilosa* reduced cecal damage as evidenced by increased hemorrhage, villus destruction and decreased villus-to-crypt ratio in chicken ceca. We also performed pyrosequencing of the PCR ampilcons based on the 16S rRNA genes of gut bacteria in chickens. Metagenomic analysis indicated that the chicken gut bacteria belonged to 6 phyla, 6 classes, 6 orders, 9 families, and 8 genera. More importantly, we found that *B*. *pilosa* affected the composition of bacteria. This change in bacteria composition was correlated with body weight gain, feed conversion ratio and gut pathology in chickens. Collectively, this work suggests that *B*. *pilosa* has beneficial effects on growth performance and protozoan infection in chickens probably via modulation of gut bacteria.

## Introduction

It is estimated that 50 billion chickens are raised in the world, reaching a global market value of 60 billion American dollars [1,2]. Chicken meat accounts for 30% of protein food consumed by humans [2].

Gut health determines growth performance and health in chickens because the gastrointestinal tract, the main digestive and absorption organ, can take in nutrients for growth and development, eliminate unwanted waste, and confer mucosal immunity against parasites [3]. A diverse microbiota is found throughout the digestive tract and is more profound in the cecum [3–6]. Gut microbiota affects nutrition, detoxification, growth performance, and protection against pathogens in chickens. Therefore, gut microbiota are important for gut health and diseases in chickens [7–9]. For instance, the absence of normal microbiota in the cecum has been considered a major factor in the susceptibility of chicks to bacterial infection [10]. Gut microbiota in chickens are gradually established from the mother and environment, including *Streptococcus* and enterobacteria within days, *Escherichia coli* and *Bacteroides* within a week [6], *Lactobacillus*, with a *Bifidobacterium* population, at an older age and finally, *Clostridium*, *Salmonella*, and *Campylobacter*. Avian coccidiosis is a bane to the poultry industry [11], causing high mortality, poor growth and high medical costs. The genus *Eimeria* (Coccidia subclass) is the causative protozoan parasite in chickens. Moreover, bacterial infections are frequently accompanied by coccidiosis [12–15].

Plants have been an extraordinary source of medicines for humans and animals since antiquity [16]. Edible plants and their compounds have become an alternative approach to treat intestinal parasites [17,18]. Further, this herbal approach can reduce or replace the abuse and misuse of antibiotics in chickens and help organic chicken production. *B*. *pilosa* (Asteraceae) is used as a medicinal plant for gastrointestinal disease, protozoan diseases and infections [16]. More recently, it was demonstrated to act against coccidiosis in chickens [19]. In this study, we extended our studies on the effect of *B*. *pilosa* on growth performance, gut microbiota and gut pathology in the presence or absence of *E*. *tenella* infection in chickens.

## Materials and Methods

### Preparation of chicken diets

Chicken diets were mixed with phosphate-buffered saline (PBS) vehicle or 0.5% *B*. *pilosa* (Chun-Yueh Biotech Company, Taiwan). Preparation of *B*. *pilosa* was processed as previously published [19]. Briefly, whole plant of *B*. *pilosa* was authenticated, processed and mixed with chicken feed.

### Animal husbandry

**Fig 1:**
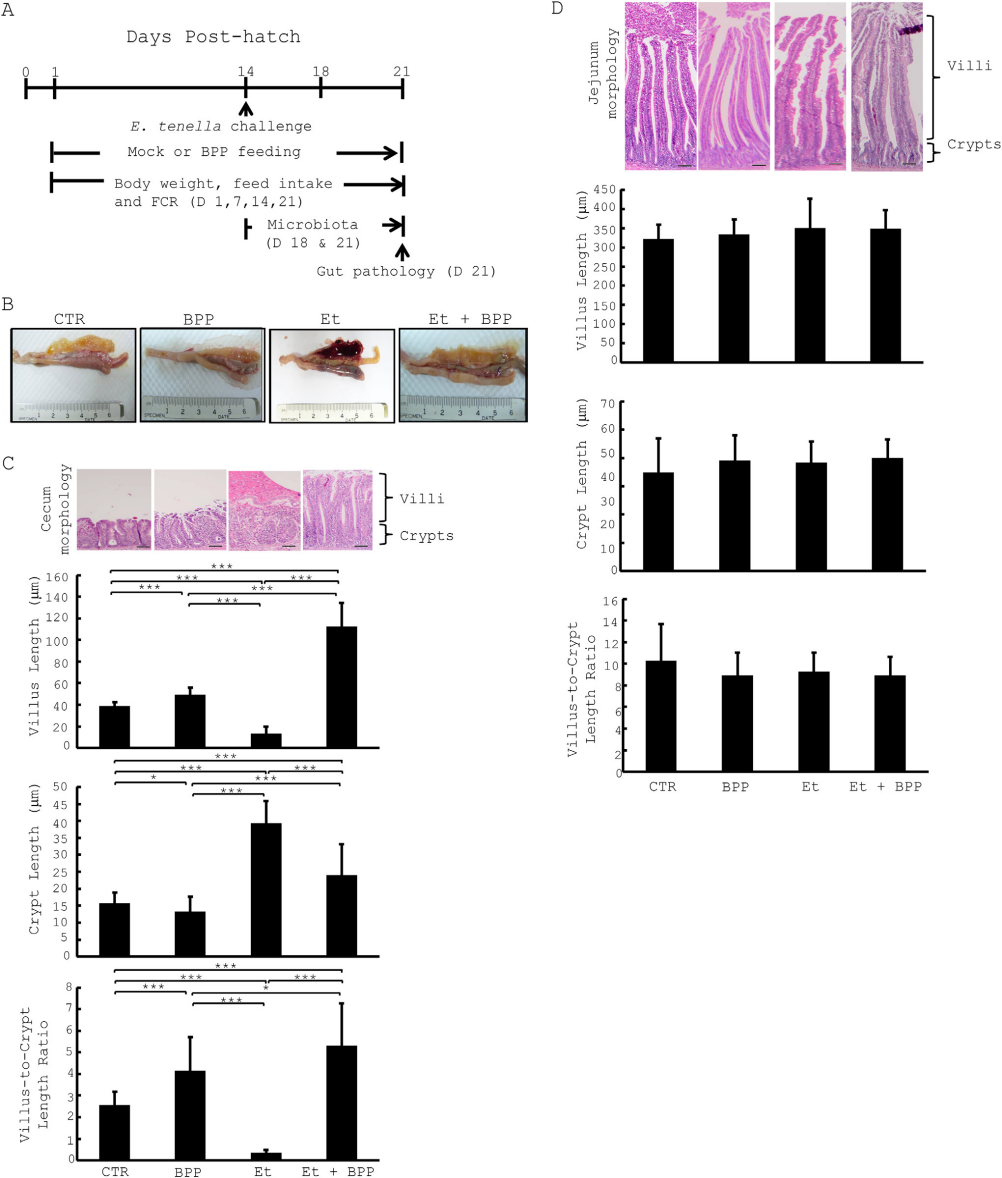
*B*. *pilosa* alleviates *E*. *tenella*-mediated gut pathology in chickens. (A) Summary of experimental protocol for this study. (B) Representative images of the gut of 21-day-old chickens in each group are shown. (C-D) Hematoxylin and esosin (H&E) staining of the ceca (C) and jejuna (D) of the same chickens as (B).

One-day-old disease-free 40 Lohmann layer chicks were purchased from a local hatchery in Taichung, Taiwan. The birds were randomly divided into 4 groups. Each group was housed in three cages: Group 1 (3, 3, 4 chicks), Group 2 (3, 3, 4 chicks), Group 3 (3, 3, 4 chicks), and Group 4 (3, 3, 4 chicks). The chicks in all the cages had free access to feed and water throughout the experiment. As shown in Fig 1A, chickens in Group 1 (uninfected untreated control chickens, CTR) and Group 2 (*E*. *tenella*-infected untreated chickens, Et) were fed with a standard diet whereas chickens in Group 3 (*B*. *pilosa* product-treated chickens, BPP) and Group 4 (*E*. *tenella*-infected *B*. *pilosa* product-treated chickens, Et + BPP) were fed with a standard diet containing 0.5% *B*. *pilosa* powder (5 g BPP/kg diet) from day 1 to day 21. On day 14, Groups 2 and 4 were infected with *E*. *tenella*. The birds were monitored once per day and raised in an institutional chicken house at 28–30°C and handled according to the guidelines of the National Chung-Hsing University Institutional Animal Care and Use Committee. The protocol was approved by the same committee (Permit Number: 103–34). Butorphenol at a dose of 1mg/kg was used to minimize suffering of the birds infected with *E*. *tenella* and all the birds were sacrificed using carbon dioxide euthanasia.

### Preparation and inoculation of *E*. *tenella* oocysts

*E*. *tenella* strain Et C1 was maintained, amplified and used throughout the experiment as previously described [19]. The oocysts were isolated from fresh feces of chickens, followed by sporulation with potassium dichromate. Four groups of birds, supplied with standard diets and standard diets containing 0.5% *B*. *pilosa* powder were tube-fed with 2 ml of sterile water (uninfected groups) or *E*. *tenella* sporulated oocysts (1 × 10^4^, infected groups) [19].

### Measurement of body weight, food conversion and gut pathology in animals

Each group of birds was individually weighed on a daily basis. Their diet consumption was monitored daily. Feed conversion ratio (FCR) was obtained by the normalization of diet consumed by body weight. To evaluate gut pathology, the ceca and intestines removed from each group of sacrificed chickens were fixed with formalin and embedded with paraffin. The gut slides were stained with hematoxylin and eosin (H&E) and examined under a microscope as described previously [19].

### Pyrosequencing and data analysis

The gut bacterial DNA collected from the feces of the chickens in Groups 1 to 4 on day 18 or 21 (i.e., day 4 and 7 post infection) were purified and used as templates for PCR amplification with 16S rRNA primers. Following pyrosequencing (Roche 454), chimeric sequences of the 16S rRNA sequences were removed using Chimera Check [20]. The trimmed sequences over 300 bp were analyzed using RDPipeline as published [21]. Briefly, 16S rRNA gene sequence alignment (Aligner), 16S rRNA gene sequence clustering (Complete Linkage Clustering), α-diversity index (Shannon Index and Chao1 estimator), rarefaction curve, and phylogenetic analysis (RDP classifier) were conducted. Principle component analysis and clustering analysis for bacterial genera were performed using the prcomp, heatmap3 and ggplot2 functions in R (the R Foundation for Statistical Computing). The hierarchical multi-level pie charts of bacterial compositions of experimental samples based on phylogenetic classifications were constructed using KRONA software [22].

### Statistical analysis

Data from 10 chickens in each group of chickens are presented as mean ± standard error (SE). Two-way ANOVA in the SPSS software was used to determine whether there were significant effects of BPP and *E*. *tenella* on chicken growth performance. Spearman's rank correlation coefficient (*r*) was computed using the cor. test function in R and used to test the association between microbiota and gut pathology and growth performance. For all statistical analyses, the *P* values less than 0.05 are considered to be statistically significant.

## Results

### *B*. *pilosa* improves growth performance and lowers FCR in control chickens and those infected with *E*. *tenella*

A previous publication showed that *B*. *pliosa* is a promising feed additive for coccidiosis control in chickens [19]. Here, we evaluated the benefits of this plant for growth performance in chickens. We first monitored the body weight gain and FCR of chickens fed with a standard diet containing vehicle and 0.5% BPP (Table 1). We found that chickens fed with *B*. *pilosa* had better body weight gain than those with a standard diet (Groups 1 and 3, Table 1). Consistently, *B*. *pilosa* significantly decreased FCR in chickens (Groups 1 and 3, Table 1). Next, we assessed the effect of *B*. *pliosa* on body weight gain and FCR in the chickens infected with *E*. *tenella*. We found that *B*. *pilosa* significantly augmented body weight gain and reduced FCR (Groups 2 and 4, Table 1). The data collectively demonstrated that *B*. *pilosa* promoted weight gain and diminished FCR in the presence or absence of *E*. *tenella* infection.

**Table 1:** Effects of *B*. *pilosa* on body weight of chickens before and after *E*. *tenella* challenge.

Group^1^	BPP	*E*. *tenella*	Day 14–21	
	(% of feed)	(oocysts)	Body weight gain (%)^2^	FCR^3^
**1(CTR)**	0	0	48.8±3.2	3.3.14±0.27
**2(Et)**	0	1×10^4^	31.6±11.5	5.16±1.23
**3(BPP)**	0.5	0	52.0±1.6	2.81±0.08
**4(BPP+Et)**	0.5	1×10^4^	45.5±5.8	3.71±0.64
***P* value**^4^	BPP		0.0046	0.0030
	*E*. *tenella*		0.0003	<0.0001
	BPP × *E*. *tenella*		0.0453	0.0481

^1^The chickens were given standard diet (Groups 1 and 2) and standard diet supplemented with 0.5% *B*. *pilosa* product (Groups 3 and 4) from days 1 to 21. On day 14, chickens in Groups 2 and 4 were orally inoculated with *E*. *tenella* at the dose of 1 × 10^4^ sporulated oocysts per chicken.

^2^Body weight gain (%) was calculated based on the formula: 100% × (body weight on day 21 minus body weight on day 14)/body weight on day 14.

^3^FCR stands for feed conversion ratio and it was obtained by normalization of feed intake to body weight gain.

^4^The influence of BPP and *E*. *tenella* treatments on body weight gain (%) and FCR in chickens were analyzed by two-way ANVOA. The *P* values less than 0.05 are considered to be statistically significant.

### Effect of *B*. *pilosa* on gut pathology associated with *E*. *tenella* infection

Next, we checked the effect of gut pathology in 4 groups of chickens. Gross examination showed that the ceca of the chickens fed with standard diet and the diet containing *B*. *pilosa*, without *E*. *tenella* infection, appeared to be similar (CTR and BPP, Fig 1B). Microscopy showed that *B*. *pilosa* seemed to have longer villus length, shorter crypt length and, in turn, higher villus-to-crypt ratio in chicken ceca (CTR and BPP, Fig 1C). However, no difference in structure of villi and crypts in the jejuna of chickens fed with and without *B*. *pilosa* was observed (CTR and BPP, Fig 1D). Further, we examined the gut pathology in the chickens infected with *E*. *tenella*. We found that the ceca of the chickens infected with *E*. *tenella* were damaged with hemorrhaging and loss of cecal villi, 7 days post *Eimeria* infection (Et, Fig 1B). Accordingly, microscopic examination indicated that *E*. *tenella* destroyed villi, increased crypt length and, in turn, reduced the villus-to-crypt ratio in chicken ceca (Et, Fig 1C), but not in chicken jejuna (Et, Fig 1D). In contrast, *B*. *pilosa* reversed the damage caused by *E*. *tenella* and, therefore, increased villus length, decreased crypt length and augmented the villus-to-crypt ratio in chicken ceca (Et + BPP, Fig 1C). Overall the data showed that *B*. *pilosa* reduced *E*. *tenella*-dependent damage in chickens via gut modulation.

### Overview of chicken gut microbiota in 8 experimental settings

**Fig 2:**
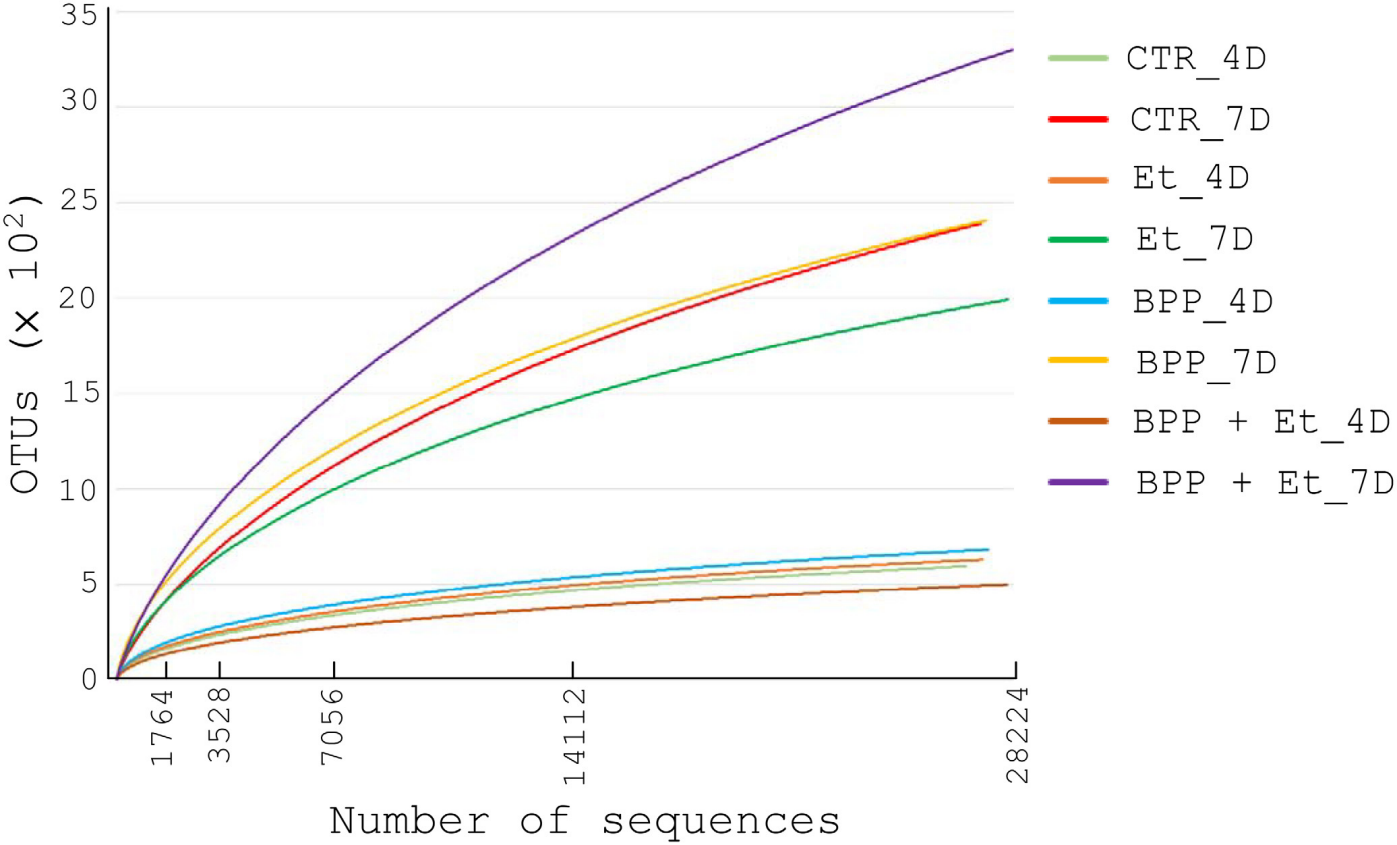
Rarefaction curves of bacterial OTUs in experimental samples from the guts of chickens fed with or without *B*. *pilosa*, infected with or without *E*. *tenella*. Rarefaction curves of bacterial 16S rRNA sequences from the guts of 18-day-old (4D) and 21-day-old (7D) chickens fed with or without *B*. *pilosa*, infected with or without *E*. *tenella*, as in Fig 1B, were analyzed.

Next, we analyzed the effect of *B*. *pilosa* on gut bacteria in each group of chickens. Pyrosequencing-based metagenomic analysis was conducted to uncover the bacterial communities in the guts of chickens aged 18 (D4) or 21 days (D7). A total of 200, 250 16S rRNA gene sequences were produced from 8 experimental samples. The number of sequences, operational taxonomic units (OTUs) and diversity indices for each sample are summarized in Table 2. Rarefaction curves suggested that the number of sequences from 8 experimental samples were enough to uncover major OTUs (Fig 2). The gut microbiota of 21-day-old chickens (7D samples) are much more diverse than those of 18-day-old chickens (4D samples) as evidenced by Shannon and Chao1 diversity indices in Table 2 and curves in Fig 2.

**Table 2:** Number of sequences, OTUs, classification and diversity indexes for each sample.

Sample DNA	Seq No.	OTUs	Phylum	Class	Order	Family	Genus	Shan	Chao1
**CTR_4D**	17048	595	4	9	9	14	23	4.27	823.4
**CTR_7D**	26031	2336	3	9	9	14	27	4.95	3837.2
**BPP_4D**	24305	682	4	8	9	13	21	4.20	927.8
**BPP_7D**	33008	2406	5	12	11	15	25	5.64	3563.7
**Et_4D**	22228	630	4	8	9	13	22	4.07	793.7
**Et_7D**	28806	1990	5	12	13	19	30	5.30	2874.7
**BPP + Et_4D**	20844	499	4	11	12	15	24	3.56	713.7
**BPP + Et_7D**	27980	3300	4	11	12	16	28	5.58	5077.8

OUT, Operational taxonomic unit; Shan, Shannon diversity index; Chao1, Chao1 diversity index

**Fig 3:**
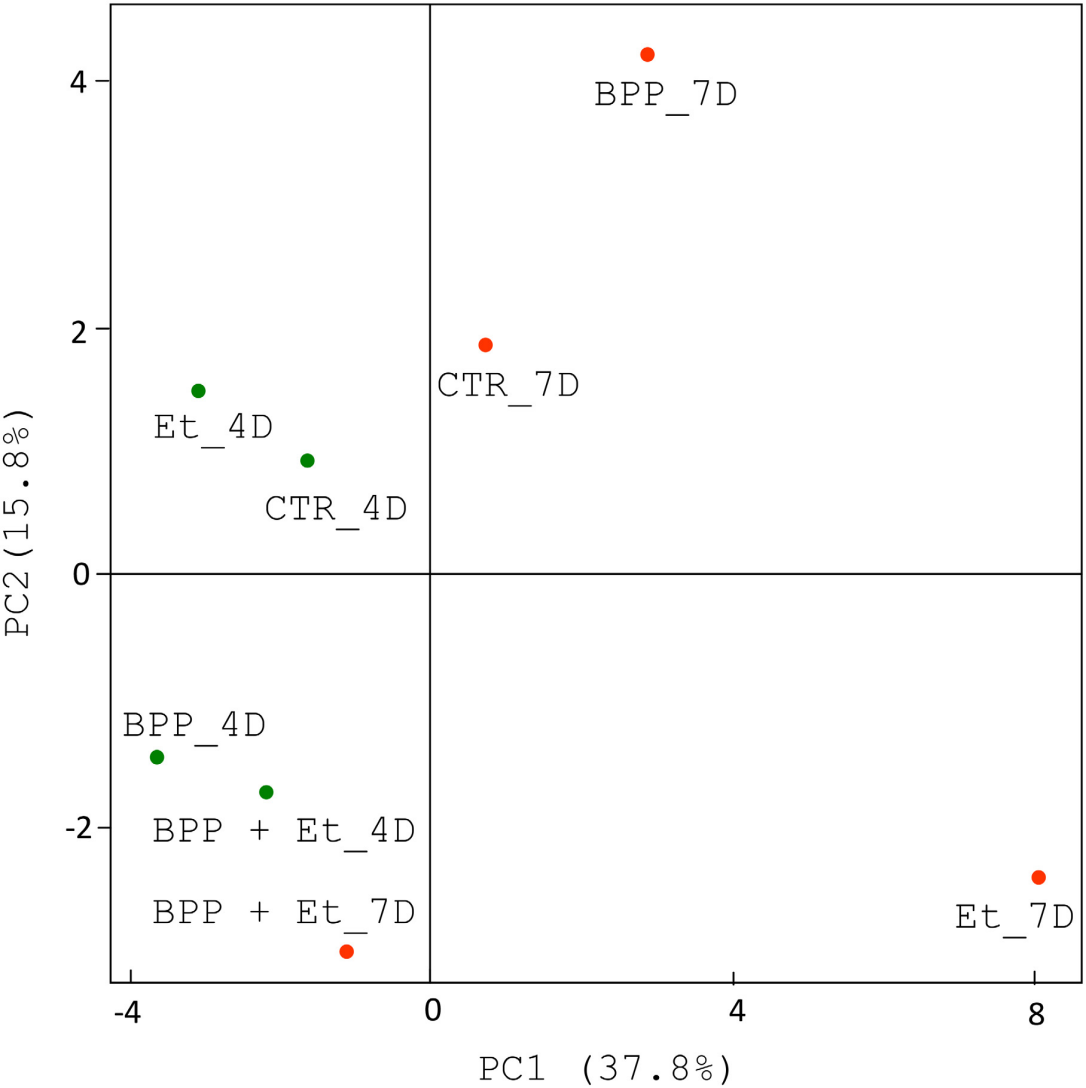
Principal component analysis of the bacterial community compositions at the genus level in the guts of chickens. Principal component analysis was conducted to compare the bacteria genera based on the 16S rRNA sequences in 8 samples as in Fig 2.

**Fig 4:**
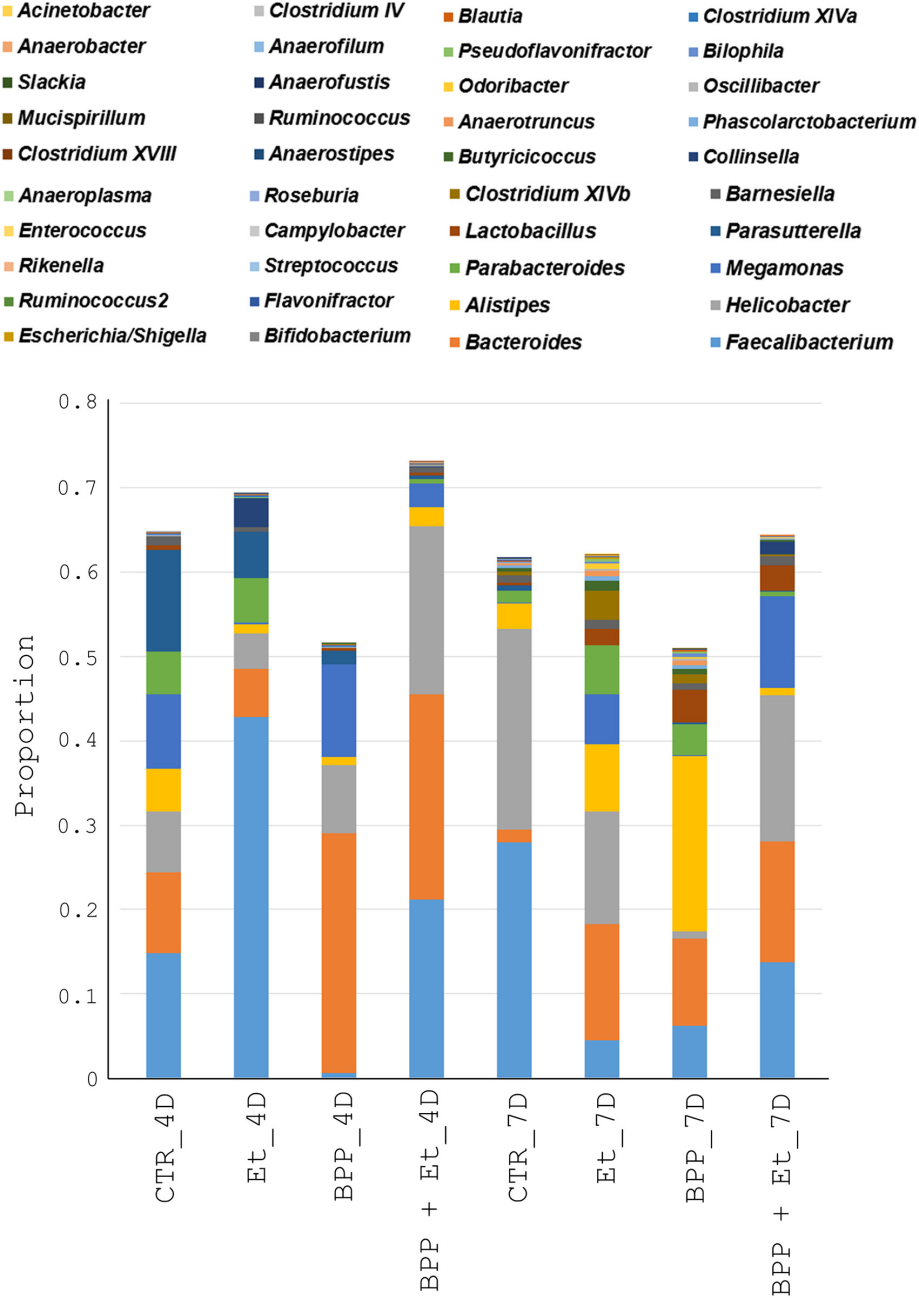
Proportion of the bacterial community compositions at the genus level in the guts of chickens. Proportion of the bacterial genera in the guts of chickens (Fig 2) was determined.

**Fig 5:**
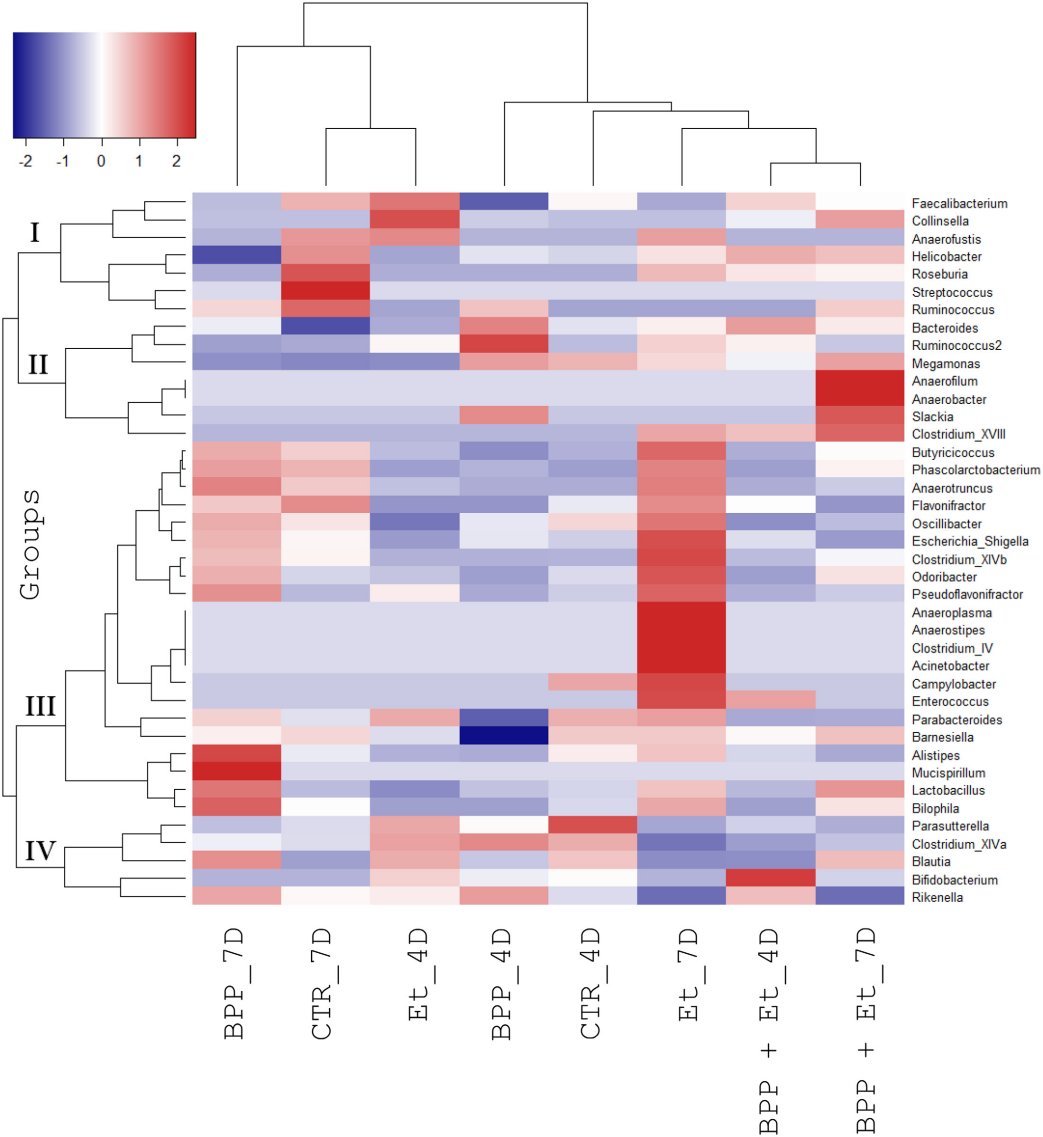
Clustering analysis of the compositions of the bacterial genera in the guts of chickens. Clustering analysis of the bacterial genera in chicken guts (Fig 2) was performed to correlate the proportion of the bacterial community with their genera.

Sequence analysis using RDP classifier revealed 6 phyla, 13 classes, 15 orders, 25 families, and 42 genera of known bacteria were present in the samples. Overall, six phyla (Firmicutes, Bacteroidetes, Proteobacteria, Actinobacteria, Tenericutes and Deferribacteres), six classes (Clostridia, Bacteroidia, Epsilonproteobacteria, Negativicutes, Bacilli and Betaproteobacteria), six orders (Clostridiales, Bacteroidales, Campylobacterales, Selenomonadales, Lactobacillales and Burkholderiales), nine families (Ruminococcaceae, Helicobacteraceae, Bacteroidaceae, Lachnospiraceae, Rikenellaceae, Veillonellaceae, Porphyromonadaceae, Lactobacillaceae and Sutterellaceae) and eight genera (*Faecalibacterium*, *Helicobacter*, *Bacteroides*, *Alistipes*, *Megamonas*, *Parabacteroides*, *Lactobacillus* and *Parasutterella*) existed in all eight samples in different proportions. Principal component analysis indicated that the bacterial community compositions of the eight samples were diverse (Fig 3). The bacterial community composition at the genus level shown in Fig 4 further confirmed the data obtained from principal component analysis. Details about the bacterial community compositions in eight samples are shown in the supplementary data (S1 Fig). To identify the co-occurring bacterial genera groups among eight experimental samples, clustering analysis was performed. As shown in Fig 5, 40 bacterial genera were grouped into four clusters. Subsets of bacterial genera associated with growth performance and *E*. *tenella* infection in chickens were identified and described below.

### Effect of *B*. *pilosa* on the change in gut microbiota

**Fig 6:**
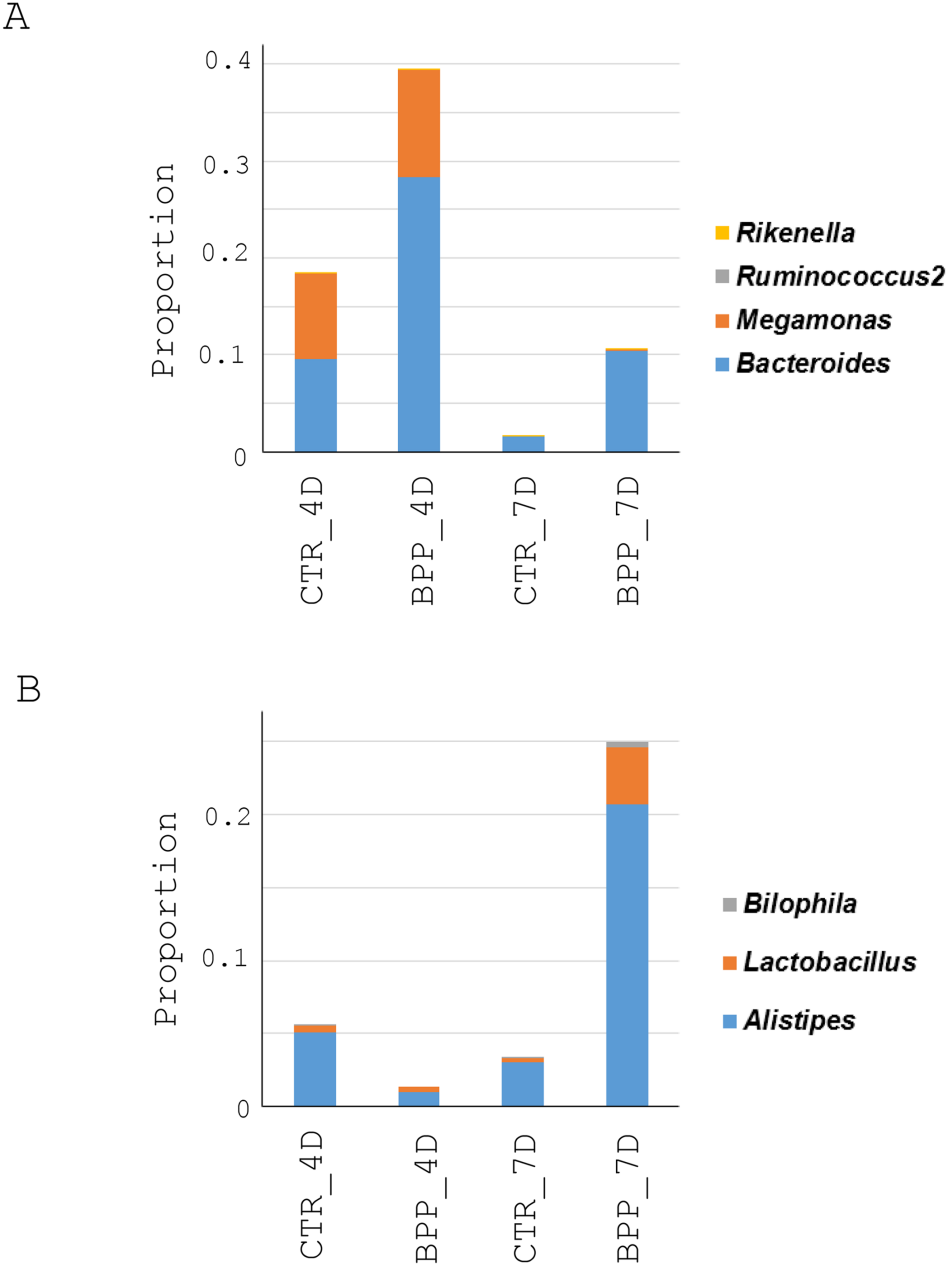
Change in the proportion of the probiotic bacterial genera in the guts of chickens fed with or without *B*. *pilosa*. The bacterial genera increased in proportion in the guts of the non-infected chickens by *B*. *pilosa* (Fig 2), were re-plotted into histograms. Only the data obtained from CTR_4D, BPP_4D, CTR_7D and BPP_7D are shown. Two patterns of change in the proportions of the bacterial genera are presented in Fig 6A and 6B. The genera in Fig 6A were increased in 18-day-old chickens whereas those in Fig 6B were increased in 21-day-old chickens.

Gut microbiota have been documented to correlate to growth performance and gut health in chickens. We therefore, first investigated the correlation between microbiota and growth performance in chickens fed with *B*. *pilosa*. Two subsets of bacterial genera in Group II and III were found to exhibit higher proportions in the guts of the chickens fed with standard diet containing *B*. *pilosa* but lower proportions in those of the other groups (Fig 5). The first subset of bacterial genera, *Bacteroides*, *Megamonas*, *Rikenella*, and *Ruminococcus2* was increased in *B*. *pilosa*-fed chickens aged 18 days (Fig 6A). Similarly, the second subset of bacterial genera, *Alistipes*, *Bilophila* and *Lactobacillus*, was elevated in *B*. *pilosa*-fed chickens aged 21 days (Fig 6B). All the above genera were reported to be beneficial microbiota. We failed to identify an elevation of these genera in the guts of chickens following *E*. *tenella* infection (data not shown) probably because the probiotics are easily disturbed by coccidiosis. Collectively, *B*. *pilosa* elevated a number of gut probiotics in chickens. Moreover, this elevation was inversely associated with FCR in chickens based on Spearman's rank correlation coefficient (*r* = −0.8 to −1).

### Gut pathology-associated bacterial genera after *E*. *tenella* infection

**Fig 7:**
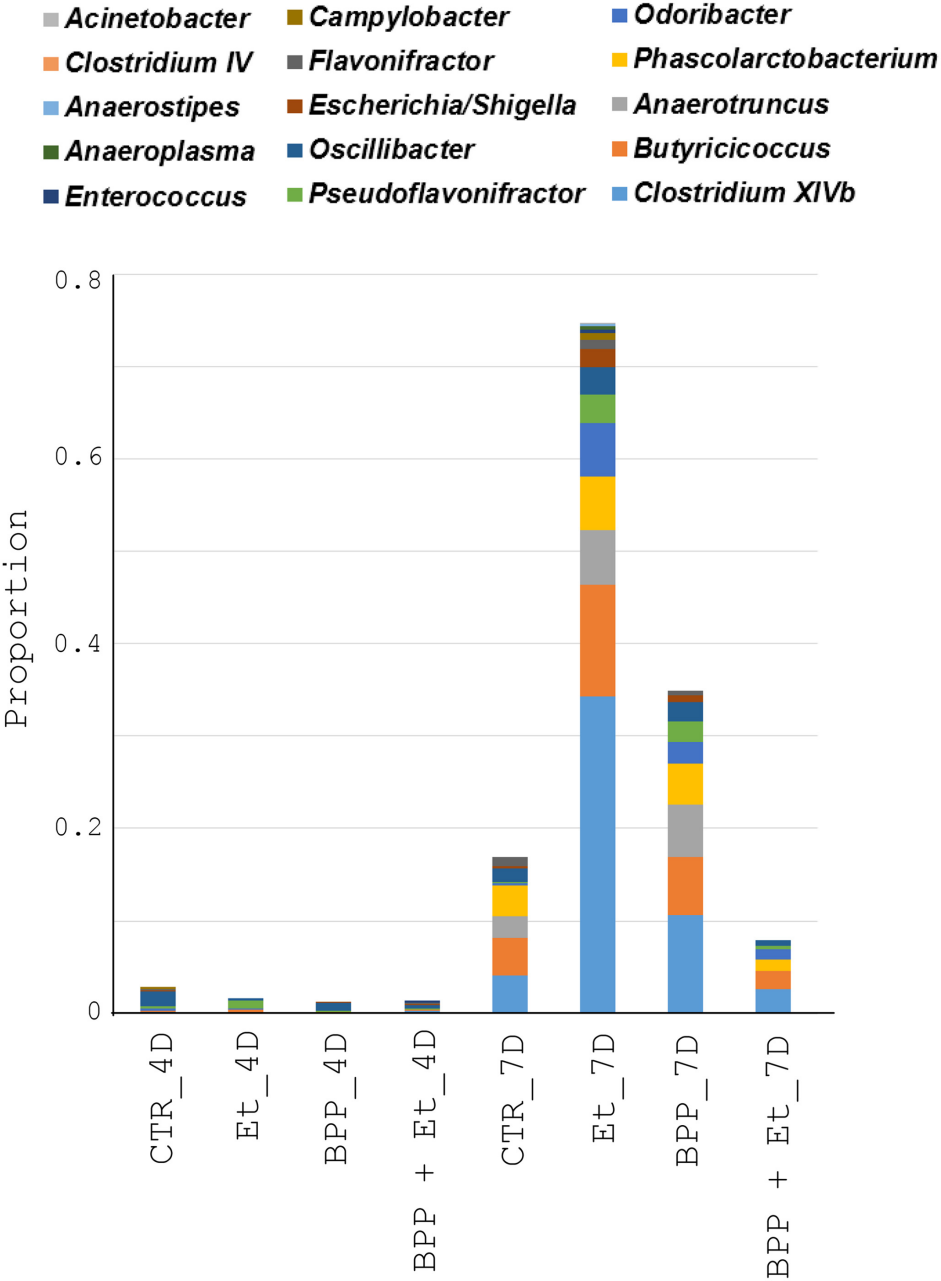
Change in the proportion of the zoonotic bacterial genera in the guts of chickens, fed with or without *B*. *pilosa*, following *E*. *tenella* infection. The bacterial genera, which decreased in proportion in the guts of the *E*. *tenella*-infected chickens by *B*. *pilosa* (Fig 2), were re-plotted into histograms.

We also wanted to correlate microbiota with gut lesions in the guts of chickens, fed with PBS and *B*. *pilosa*, following *E*. *tenella* infection. Cluster analysis showed that one subset of 15 bacterial genera in Group III exhibited higher proportions in the guts of the *E*. *tenella*-infected chickens as they aged but lower proportions in the *B*. *pilosa*-fed *E*. *tenella*-infected chickens (Et_7D vs BPP + Et_7D, Fig 5). Change in the proportion of the 15 bacterial genera in the guts of chickens, aged 18 days, 4 days post-infection was not evident (Et_4D vs BPP + Et_4D, Fig 7). However, this change became evident in *E*. *tenella*-infected chickens, aged 21 days, suggesting that the 15 bacterial genera in chicken guts were associated with gut pathology (Et_7D vs BPP + Et_7D, Fig 7). These bacteria included *Actinobacter*, *Clostridium IV*, *Anaerostipes*, *Anaeroplasma*, *Enterococcus*, *Campylobacteria*, *Flavonifractor*, *Escherichia/Shigella*, *Oscillibacter*, *PseodoFlavonifractor*, *Odoribacter*, *Phascolarctobacterium*, *Anaerotruncus*, *Butyricicoccus* and *Clostridium XIVb*. Among them, *Escherichia/Shigella*, *Campylobacter*, *Enterococcus*, *Clostridium* and *Acinetobacter* are known as opportunistic pathogens of zoonotic origin, that not only affect the domestic animal industry but also cause public health problems in humans [23–29]. Of note, *B*. *pilosa* reduced the proportion of these opportunistic zoonotic pathogens in the guts of the chickens, suggesting this plant inhibited the pathogenic bacteria in the guts of chickens infected with *E*. *tenella* (Fig 7). Moreover, the decrease in the above 5 harmful genera was inversely correlated with villus length and the villus-to-crypt ratio, hallmarks of gut pathology, in chickens based on Spearman’s rank correlation coefficient (*r* = −0.8 to −1).

## Discussion

Akin to human medicine, medicinal herbs and their compounds have been used as veterinary medicines in animals via multiple mechanisms of action. Their mechanisms include regulation of nutrient digestion and absorption, microbiota, immunity and ROS clearance, *etc*. Here, we showed that *B*. *pilosa* enhanced growth performance (Table 1), changed gut microbiota (Table 2 and Fig 5) and reduced *E*. *tenella*-implicated gut pathogenesis (Fig 1C and 1D). Besides, *B*. *pilosa* selectively increased probiotics and decreased harmful bacteria in the guts of chickens (Figs 5–7).

*B*. *pilosa* and its phytochemicals were reported to modulate gastrointestinal diseases, immunity and bacteria [16]. However, anti-coccidial compounds of *B*. *pilosa* need to be further ascertained. The mechanism by which *B*. *pilosa* ameliorates growth performance and coccidiosis in chickens is unknown. Our data collectively suggest that *B*. *pilosa* regulates a shift in gut microbiota in chickens. In fact, we found that *B*. *pilosa* did alter the proportion of gut microbiota in chickens, including an increase in 7 probiotic genera (Figs 5 and 6) and a decrease in 15 bacterial genera, including 5 harmful bacteria (Figs 5 and 7). As far as the 7 probiotics are concerned, *Alistipes*, *Bacteroides*, *Lactobacillus*, and *Ruminococcus* are known as probiotics for growth performance and weight gain in chickens [30,31]. *Bacteroides* and *Megamonas* were reported to be implicated in propionate production in chicken guts [32]. *Megamonas* and *Ruminococcus* were reported to be involved in polysaccharide degradation and utilization in chicken guts [32]. *Bacteroides* and *Lactobacillus* were shown to produce some essential vitamins (i.e., vitamin K, vitamin B12, and folic acid) and contribute to intestinal bile acid metabolism and recirculation [33]. Moreover, *Lactobacillus* has been used as a probiotic to control coccidiosis in chickens infected with *Eimeria* species [34,35]. Thus, gut microbiota play an important role in the clinical outcomes of coccidiosis in chickens [34,35]. In sharp contrast, the proportion of 15 bacterial genera in chicken guts was decreased by *B*. *pilosa* (Fig 7). Among them, *Escherichia/Shigella*, *Campylobacter*, *Enterococcus*, *Clostridium* and *Acinetobacter*, known as opportunistic pathogens of zoonotic origin, not only affect domestic animal industry but also cause public health problems in men [23–29]. Consistent with the function of *B*. *pilosa* in outgrowth of probiotics, this plant prevented growth of these opportunistic zoonotic pathogens in chicken guts (Fig 7). This study supports the notion that *B*. *pilosa* acts as a prebiotic to enhance the growth of probiotics in chicken guts to increase growth performance and reduce coccidiosis. It should be also noted that *B*. *pilosa* may be useful as a feed substituent and additive, as our results show that it can improve growth performance and coccidiosis (Table 1 and Fig 1). This application also lowers feed cost from crops and anti-coccidial agents, and risk of anti-coccidial contamination. This work also expands the medicinal utility of *B*. *pilosa* in animals [36,37], to target the balance of gut microbiota [38,39].

## Conclusions

Here, we studied and demonstrated the beneficial effect of *B*. *pilosa* on growth performance (*i*.*e*., body weight gain and FCR), gut bacteria and *E*. *tenella* infection in chickens. Overall the data suggest that *B*. *pilosa* may have a novel function as a prebiotic, which elevates beneficial bacteria and reduces harmful bacteria in chicken guts. This work further illustrates the potential use of *B*. *pilosa* as a feed additive in organic chicken production.

## Supporting Information

S1 Fig Compositions of gut bacteria present in the guts of 18- and 21-day-old chickens, fed with PBS and *Bidens pilosa*, infected with PBS and *E*. *tenella*. The chickens from Groups 1 to 4 were sacrificed on days 4 (D4) and 7 (D7) and the bacterial DNA samples of their guts (ceca and intestines) were pooled into 8 samples. Individual bacterial community composition was analyzed and listed in A (CTR_4D), B (BPP_4D), C (Et_4D), D (BPP + Et_4D), E (CTR_4D), F (BPP_4D), G (Et_4D) and H (BPP + Et_4D) from the guts of samples. *B*. *pilosa* alleviates *E*. *tenella*-mediated gut pathology in chickens.
(PDF)

## Abbreviations

PBS: phosphate-buffered saline
OTU: operational taxonomic unit
CTR: uninfected untreated control
Et: *Eimeria tenella*-infected untreated
BPP: *B*. *pilosa* product-treated
Et + BPP: *Eimeria tenella*-infected *B*. *pilosa* product-treated
FCR: Feed conversion ratio
H&E: hematoxylin and eosin
OUT: Operational taxonomic unit
Shan: Shannon diversity index
Chao1: Chao1 diversity index
