# Supplementary material for: Beneficial Effect of Bidens pilosa on Body Weight Gain, Food Conversion Ratio, Gut Bacteria and Coccidiosis in Chickens
Source: PLoS One. 2016 Jan 14;11(1):e0146141. doi: 10.1371/journal.pone.0146141 (PMC4713076; doi:10.1371/journal.pone.0146141)
Supplement: S1 Fig — The chickens from Groups 1 to 4 were sacrificed on days 4 (D4) and 7 (D7) and the bacterial DNA samples of their guts (ceca and intestines) were pooled into 8 samples. Individual bacterial community composition was analyzed and listed in A (CTR_4D), B (BPP_4D), C (Et_4D), D (BPP + Et_4D), E (CTR_4D), F (BPP_4D), G (Et_4D) and H (BPP + Et_4D) from the guts of samples. B. pilosa alleviates E. tenella-mediated gut pathology in chickens. (PDF) [file pone.0146141.s001.pdf]

A

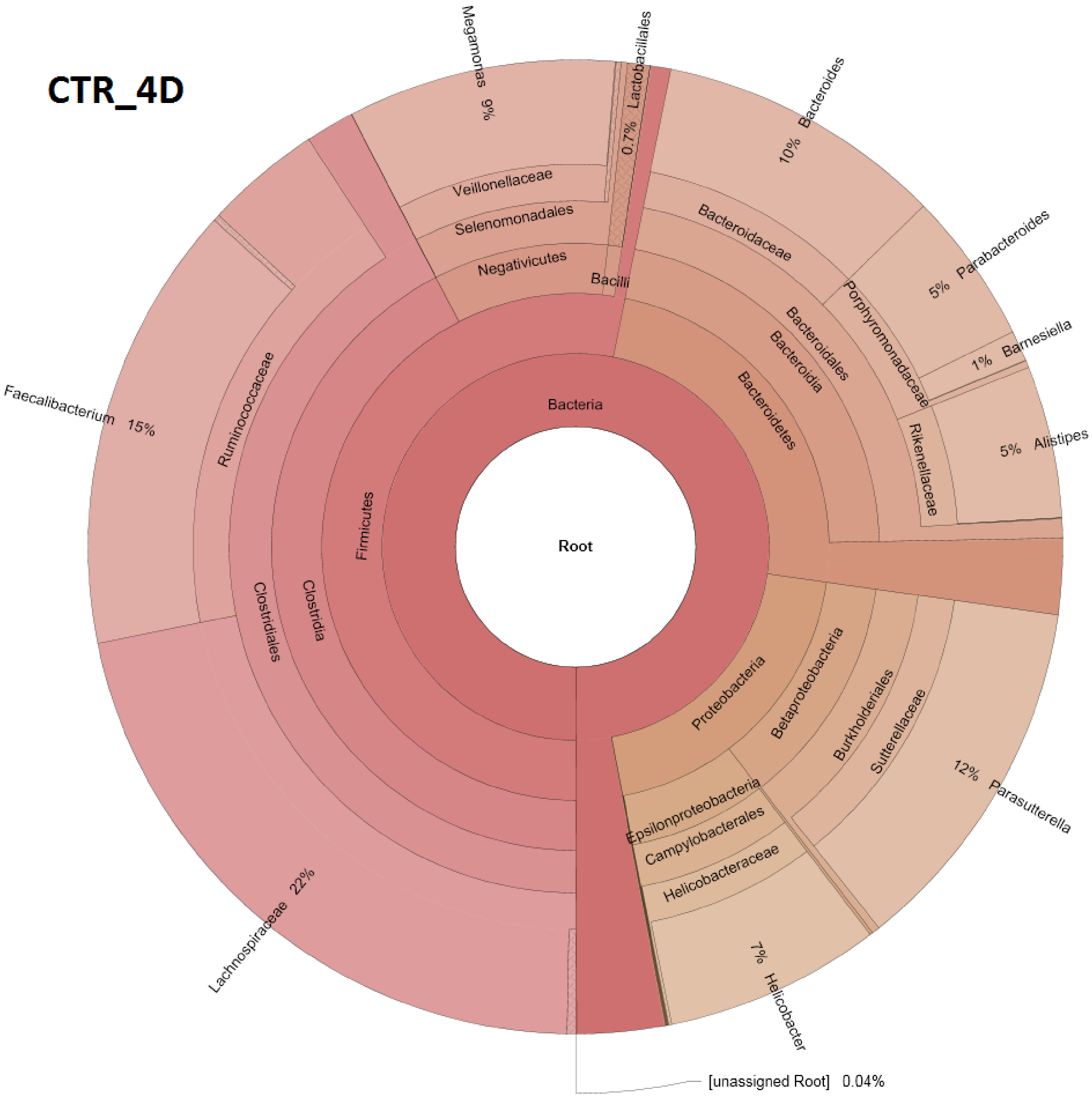
CTR_4D
Root
Bacteria
Firmicutes
Clostridia
Clostridiales
Ruminococcaceae
Faecalibacterium 15%
Lachnospiraceae 22%
Negativicutes
Selenomonadales
Veillonellaceae
Megamonas 9%
Bacilli
Lactobacillales 0.7%
Bacteroidetes
Bacteroidia
Bacteroidales
Bacteroidaceae
Bacteroides 10%
Porphyromonadaceae
Parabacteroides 5%
Barnesiella 1%
Rikenellaceae
Alistipes 5%
Proteobacteria
Betaproteobacteria
Burkholderiales
Sutterellaceae
Parasutterella 12%
Epsilonproteobacteria
Campylobacterales
Helicobacteraceae
Helicobacter 7%
[unassigned Root] 0.04%

B

BPP_4D

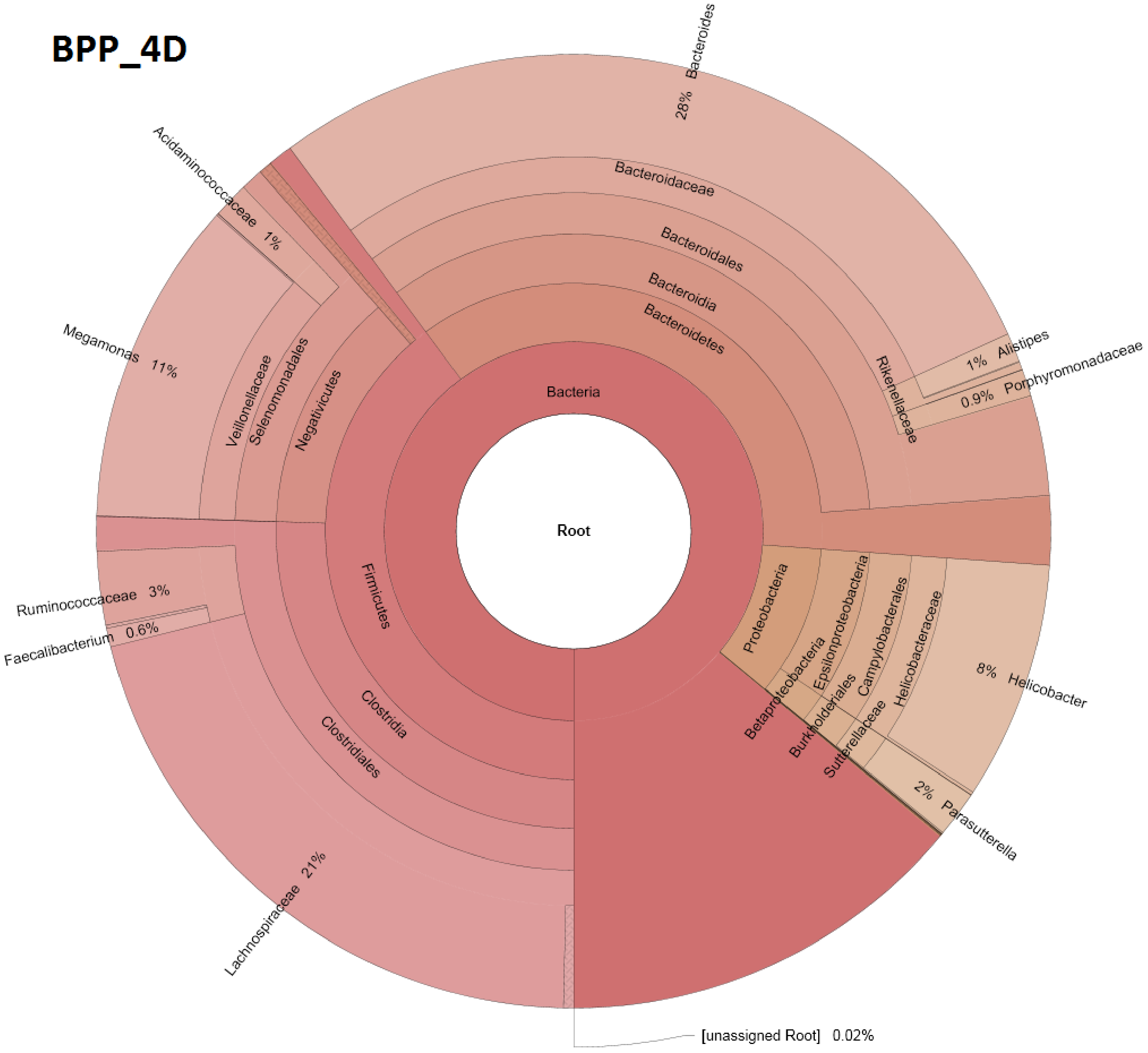
Root
Bacteria
Bacteroidetes
Bacteroidia
Bacteroidales
Bacteroidaceae
28% Bacteroides
Rikenellaceae
1% Alistipes
0.9% Porphyromonadaceae
Proteobacteria
Betaproteobacteria
Burkholderiales
Sutterellaceae
2% Parasutterella
Epsilonproteobacteria
Campylobacterales
Helicobacteraceae
8% Helicobacter
Firmicutes
Clostridia
Clostridiales
Lachnospiraceae 21%
Ruminococcaceae 3%
Faecalibacterium 0.6%
Negativicutes
Selenomonadales
Veillonellaceae
Megamonas 11%
Acidaminococcaceae 1%
[unassigned Root] 0.02%

C

Et_4D

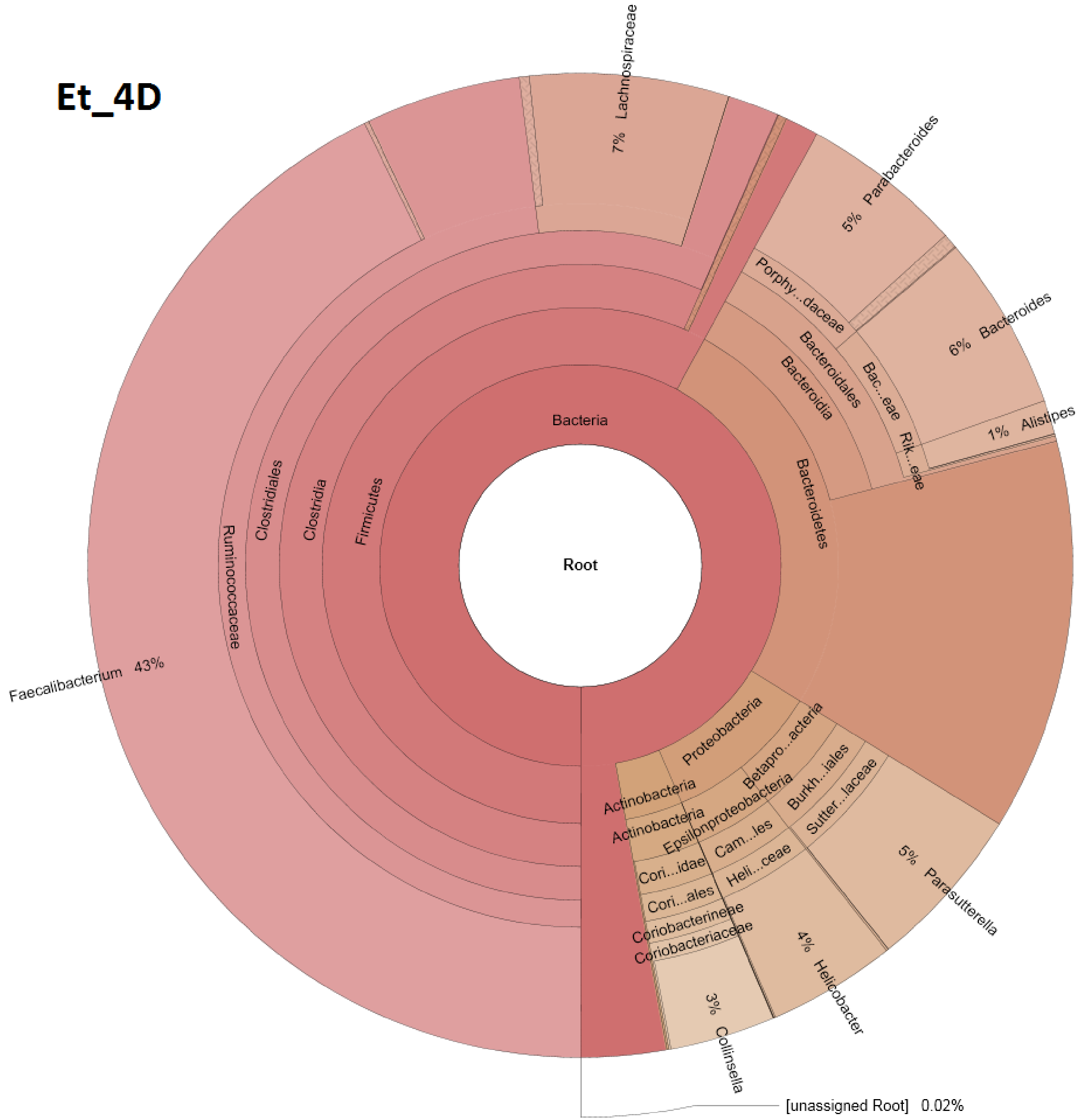

Root
Bacteria
Firmicutes
Clostridia
Clostridiales
Ruminococcaceae
Faecalibacterium 43%
7% Lachnospiraceae
Bacteroidetes
Bacteroidia
Bacteroidales
Porphy...daceae
5% Parabacteroides
Bac...eae
6% Bacteroides
Rik...eae
1% Alistipes
Proteobacteria
Betapro...acteria
Burkh...iales
Sutter...laceae
5% Parasutterella
Epsilonproteobacteria
Cam...les
Heli...ceae
4% Helicobacter
Actinobacteria
Actinobacteria
Cori...idae
Cori...ales
Coriobacterineae
Coriobacteriaceae
3% Collinsella
[unassigned Root] 0.02%

D

BPP + Et _4D

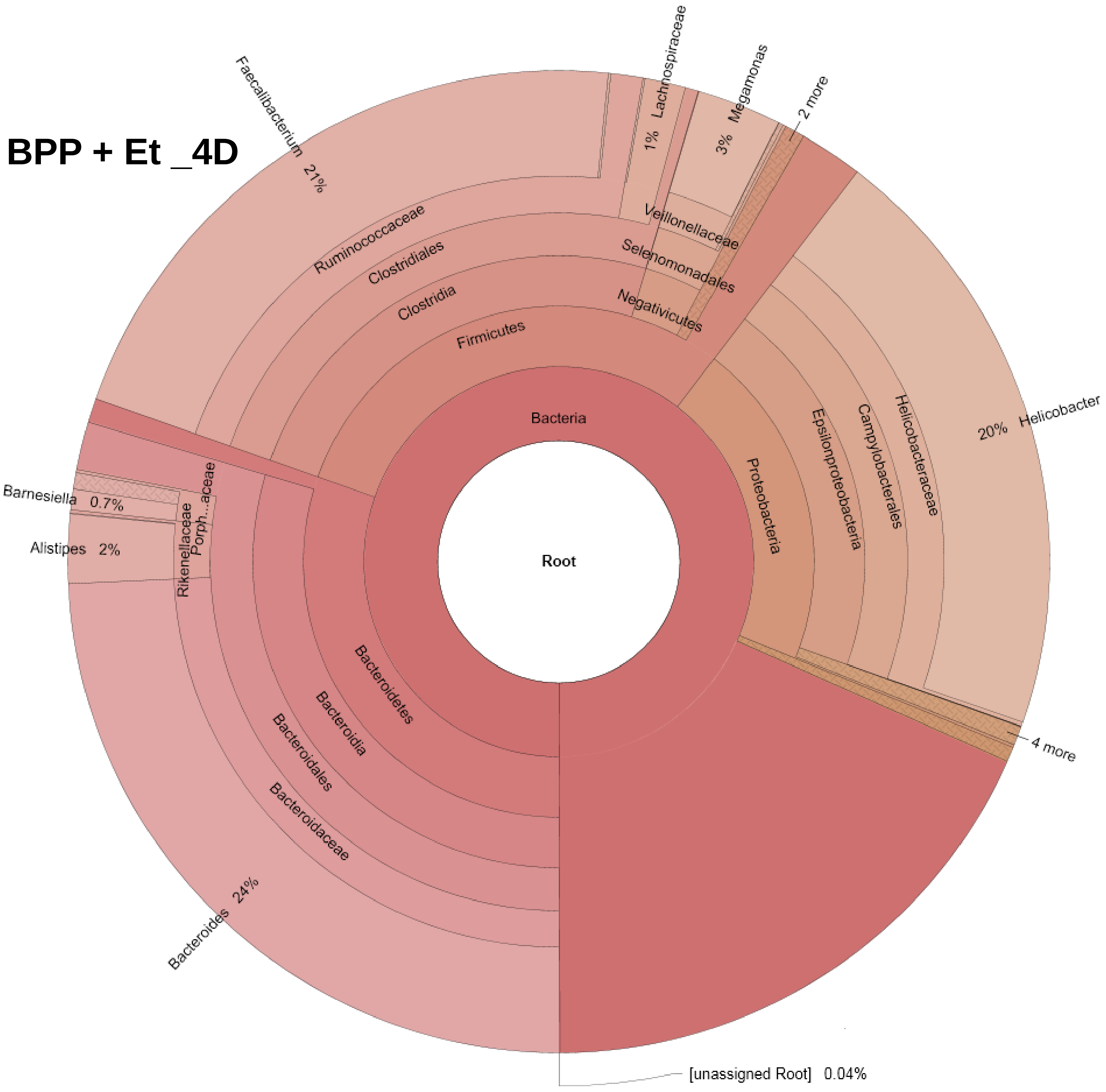

Root
Bacteria
Firmicutes
Clostridia
Clostridiales
Ruminococcaceae
Faecalibacterium 21%
Lachnospiraceae
1%
Negativicutes
Selenomonadales
Veillonellaceae
Megamonas
3%
2 more
Proteobacteria
Epsilonproteobacteria
Campylobacterales
Helicobacteraceae
20% Helicobacter
4 more
Bacteroidetes
Bacteroidia
Bacteroidales
Bacteroidaceae
Bacteroides 24%
Porph...aceae
Rikenellaceae
Barnesiella 0.7%
Alistipes 2%
[unassigned Root] 0.04%

E

CTR_7D

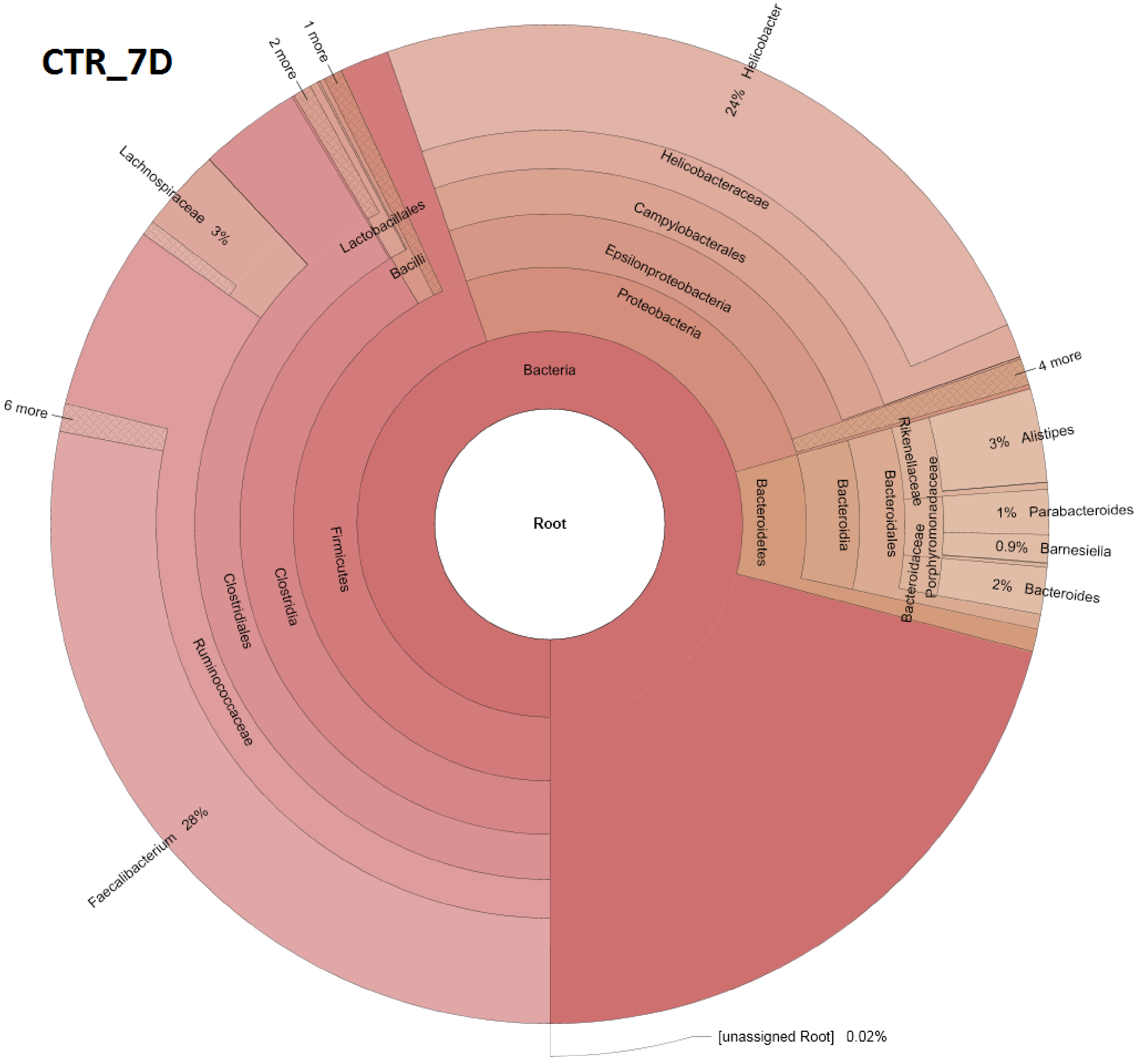

Root
Bacteria
Firmicutes
Clostridia
Clostridiales
Ruminococcaceae
Faecalibacterium 28%
Lachnospiraceae 3%
6 more
Bacilli
Lactobacillales
2 more
1 more
Proteobacteria
Epsilonproteobacteria
Campylobacterales
Helicobacteraceae
24% Helicobacter
4 more
Bacteroidetes
Bacteroidia
Bacteroidales
Rikenellaceae
Porphyromonadaceae
Bacteroidaceae
3% Alistipes
1% Parabacteroides
0.9% Barnesiella
2% Bacteroides
[unassigned Root] 0.02%

F

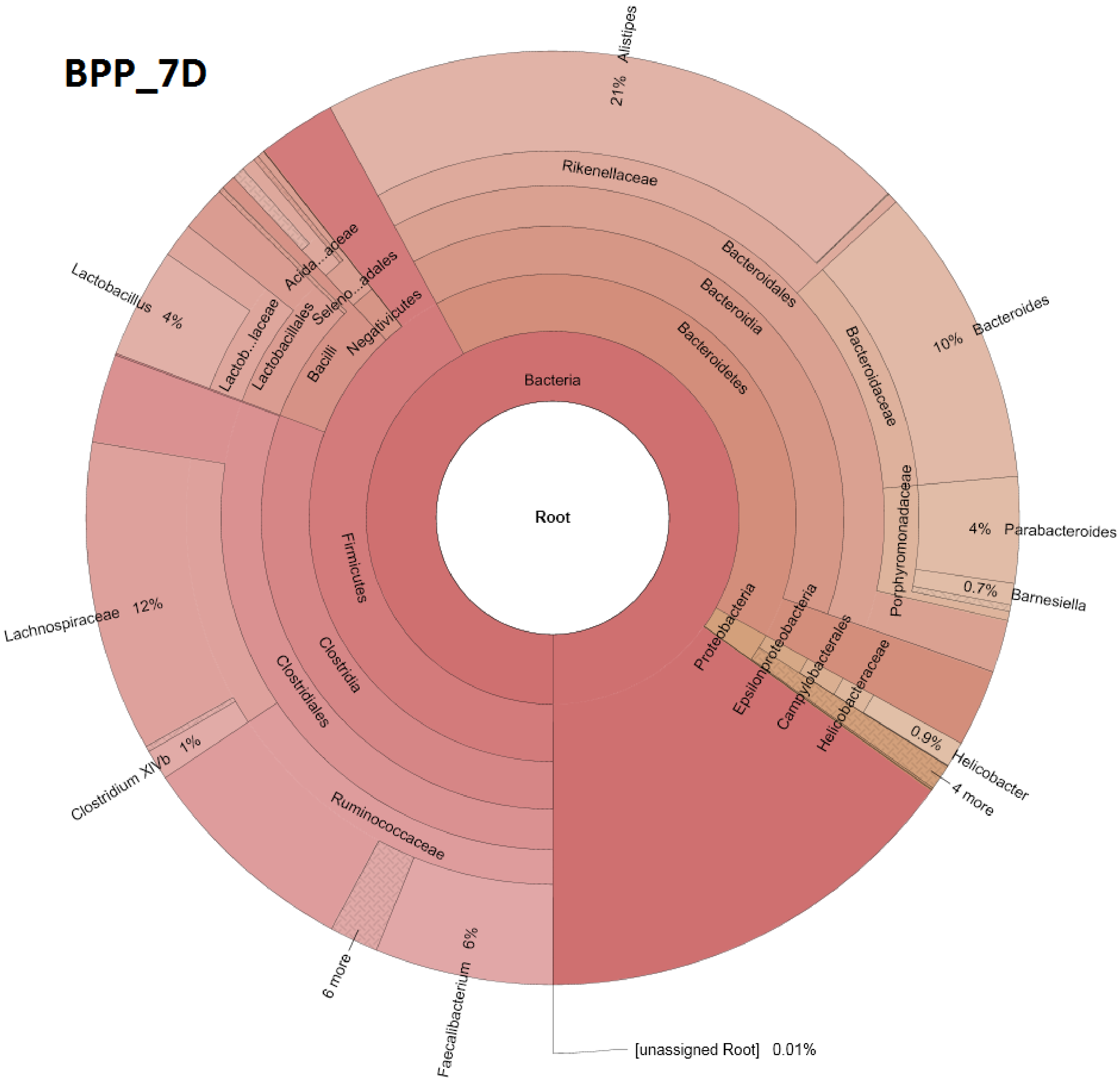
BPP_7D
Root
Bacteria
Bacteroidetes
Bacteroidia
Bacteroidales
Rikenellaceae
21% Alistipes
Bacteroidaceae
10% Bacteroides
Porphyromonadaceae
4% Parabacteroides
0.7% Barnesiella
Proteobacteria
Epsilonproteobacteria
Campylobacterales
Helicobacteraceae
0.9% Helicobacter
4 more
Firmicutes
Clostridia
Clostridiales
Lachnospiraceae 12%
Clostridium XlVb 1%
Ruminococcaceae
Faecalibacterium 6%
6 more
Bacilli
Lactobacillales
Lactob...laceae
Lactobacillus 4%
Negativicutes
Seleno...adales
Acida...aceae
[unassigned Root] 0.01%

G

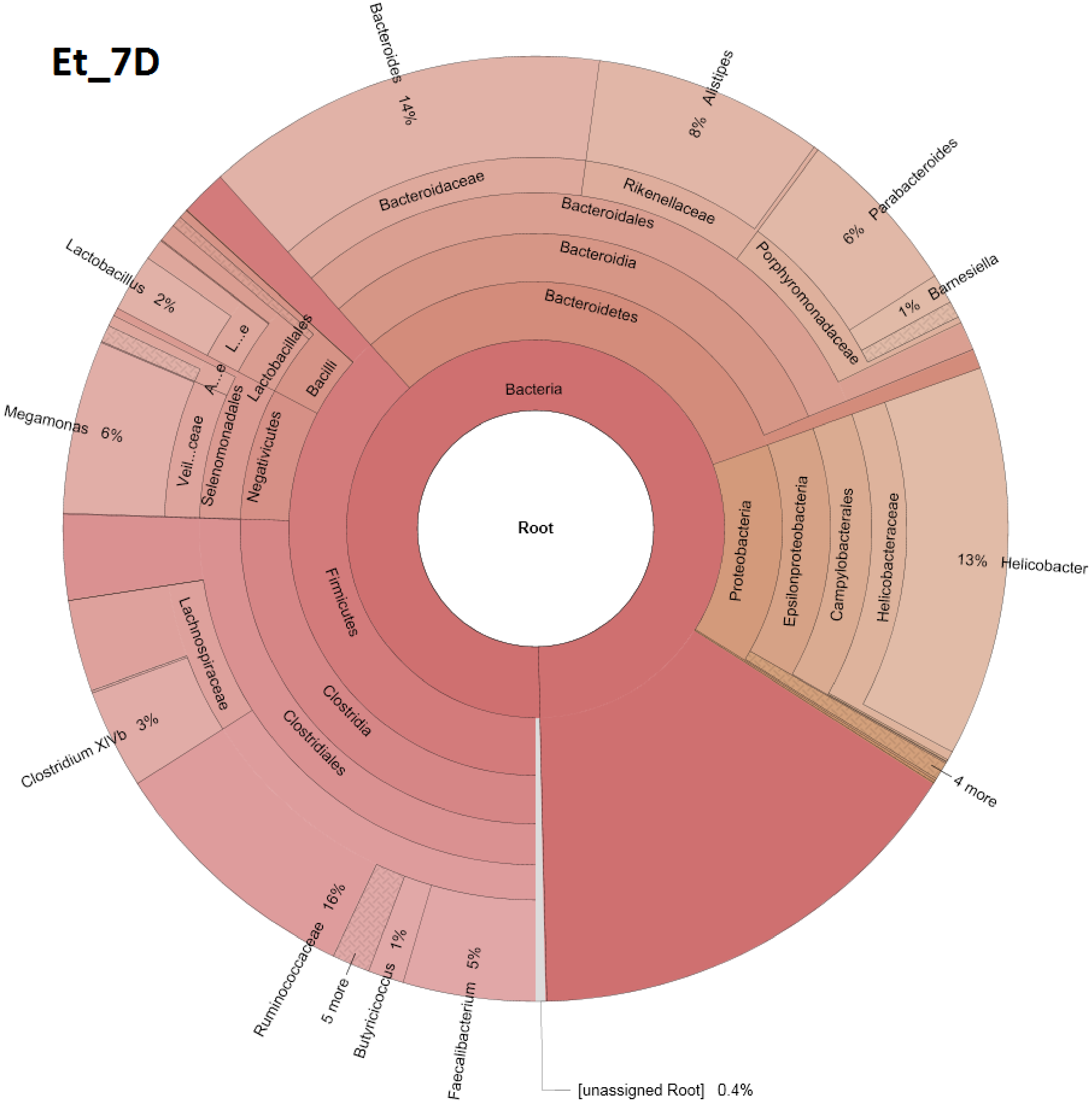
Et_7D
Root
Bacteria
Bacteroidetes
Bacteroidia
Bacteroidales
Bacteroidaceae
Bacteroides 14%
Rikenellaceae
Alistipes 8%
Porphyromonadaceae
Parabacteroides 6%
Barnesiella 1%
Proteobacteria
Epsilonproteobacteria
Campylobacterales
Helicobacteraceae
Helicobacter 13%
4 more
Firmicutes
Bacilli
Lactobacillales
L...e
Lactobacillus 2%
Negativicutes
Selenomonadales
A...e
Veil...ceae
Megamonas 6%
Clostridia
Clostridiales
Lachnospiraceae
Clostridium XIVb 3%
Ruminococcaceae 16%
5 more
Butyricicoccus 1%
Faecalibacterium 5%
[unassigned Root] 0.4%

H

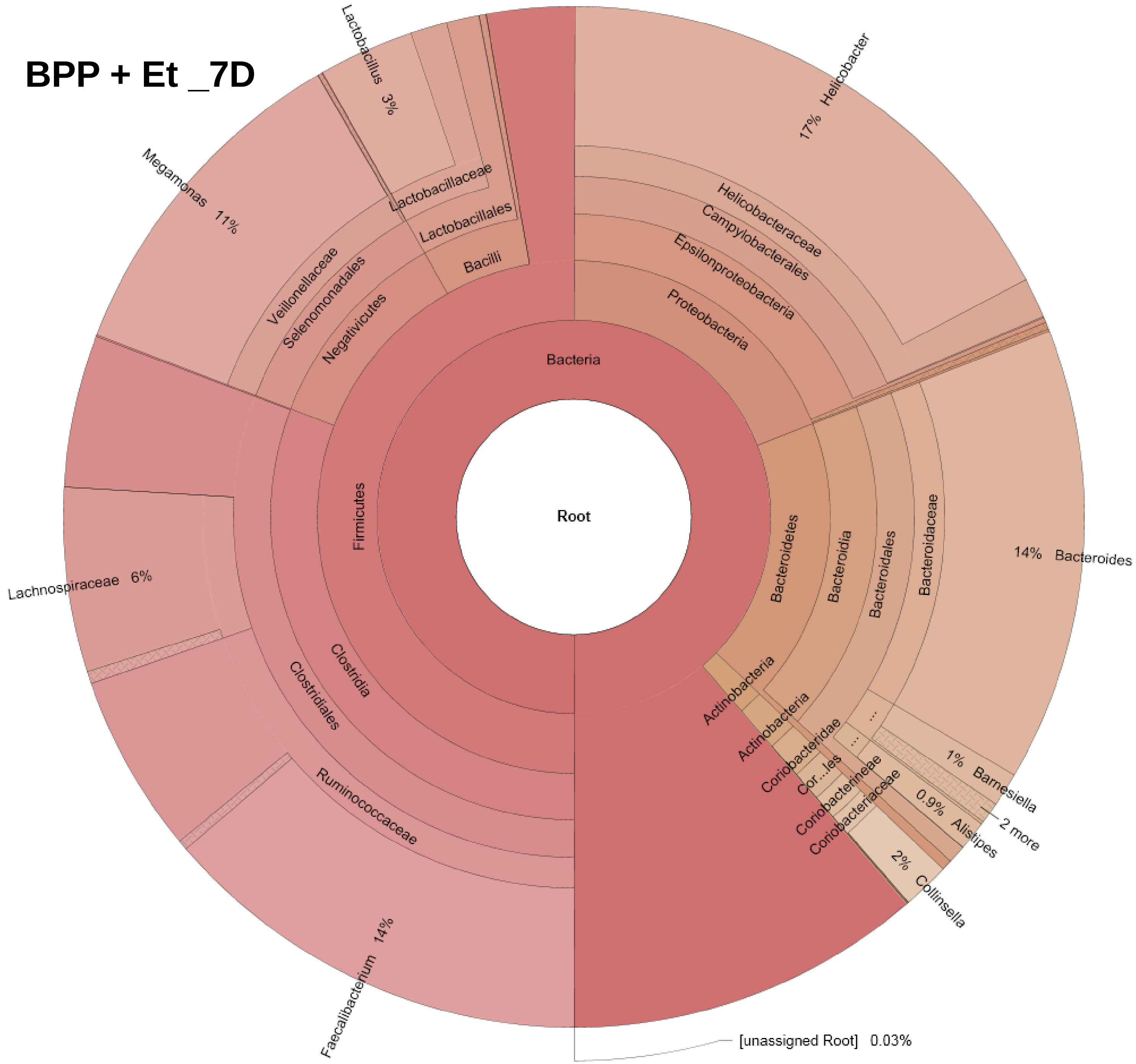
BPP + Et _7D
Root
Bacteria
Firmicutes
Bacilli
Lactobacillales
Lactobacillaceae
Lactobacillus 3%
Negativicutes
Selenomonadales
Veillonellaceae
Megamonas 11%
Clostridia
Clostridiales
Lachnospiraceae 6%
Ruminococcaceae
Faecalibacterium 14%
Proteobacteria
Epsilonproteobacteria
Campylobacterales
Helicobacteraceae
17% Helicobacter
Bacteroidetes
Bacteroidia
Bacteroidales
Bacteroidaceae
14% Bacteroides
1% Barnesiella
2 more
0.9% Alistipes
Actinobacteria
Actinobacteria
Coriobacteridae
Cor...les
Coriobacterineae
Coriobacteriaceae
2% Collinsella
[unassigned Root] 0.03%

S1 Fig. Compositions of gut bacteria present in the guts of 18- and 21-day-old chickens, fed with PBS and *Bidens pilosa*, infected with PBS and *E. tenella*. The chickens from Groups 1 to 4 were sacrificed on days 4 (D4) and 7 (D7) and the bacterial DNA samples of their guts (ceca and intestines) were pooled into 8 samples. Individual bacterial community composition was analyzed and listed in S1A (CTR_4D), S1B (BPP_4D), S1C (Et_4D), S1D (BPP + Et_4D), S1E (CTR_4D), S1F (BPP_4D), S1G (Et_4D) and S1H (BPP + Et_4D) from the guts of samples. *B. pilosa* alleviates *E. tenella*-mediated gut pathology in chickens. (A) Summary of experimental protocol in this study. (B) Representative images of the guts of chickens in each group are shown. (C-D) H&E staining of the ceca (C) and jejuna (D) of the same chickens as (B).
